# The Effect of a School-Based Intervention on Physical Activity and Well-Being: a Non-Randomised Controlled Trial with Children of Low Socio-Economic Status

**DOI:** 10.1186/s40798-018-0129-0

**Published:** 2018-04-20

**Authors:** Stephen Shannon, Deirdre Brennan, Donncha Hanna, Zoe Younger, Jessica Hassan, Gavin Breslin

**Affiliations:** 1 Sport and Exercise Sciences Research Institute, Ulster University, Jordanstown, BT37 OQB Northern Ireland; 2 School of Psychology, Queen’s University Belfast, Belfast, BT9 5BN Northern Ireland; 3 The Bamford Centre for Mental Health and Well-being, Belfast, BT15 1ED Northern Ireland

**Keywords:** Health promotion, Behaviour change, Needs satisfaction, Motivation, Physical education

## Abstract

**Background:**

Self-determination theory (SDT) has been used to predict children’s physical activity and well-being. However, few school-based SDT intervention studies have been conducted, and no research exists with children of low socio-economic status (SES). Therefore, SDT-derived needs-supportive teaching techniques informed the design and analyses of the Healthy Choices Programme (HCP). The aim was to determine if the HCP could enhance moderate-to-vigorous physical activity (MVPA) and well-being among children of low SES through increasing autonomy-support, needs satisfaction and intrinsic motivation.

**Method:**

A mixed factorial two (group) × two (time) wait-list controlled trial was conducted and reported using the TREND guidelines. A total of 155 children (56% females; intervention *n* = 84, control *n* = 71) took part and completed measures at baseline (week 0) and post-intervention (week 11). The effect of the intervention on MVPA (model 1) and well-being (model 2) was tested through serial mediation models with three mediators (i.e. autonomy-support, needs satisfaction and intrinsic motivation).

**Results:**

In comparison to the control group, the intervention was related to increases in MVPA (*β* = .45) and autonomy-support (*β* = .17). In model 1, analyses revealed partial mediation of the MVPA change through autonomy-support (*β* = .14), intrinsic motivation (*β* = .51) and all three SDT mediators in sequence (total *r*^*2*^ = .34). In model 2, well-being was indirectly enhanced through autonomy-support (*β* = .38) and autonomy-support and needs satisfaction in sequence (total *r*^*2*^ = .21).

**Conclusions:**

The HCP enhanced MVPA and well-being by engendering a needs-supportive physical activity environment. The scientific and practical contribution of this study was the application of SDT in all aspects of the HCP intervention’s design and analyses. Practitioners may consider integrating SDT principles, as implemented in the HCP, for health promotion.

**Trial Registration:**

This study is registered on Research Registry (number researchregistry2852).

**Electronic supplementary material:**

The online version of this article (10.1186/s40798-018-0129-0) contains supplementary material, which is available to authorized users.

## Key points

- A self-determination theory-based intervention had a positive impact on children’s moderate-to-vigorous physical activity and well-being.
- Well-being and physical activity were enhanced through the children perceiving greater autonomy-support from their teachers, psychological needs satisfaction and intrinsic motivation.
- Practitioners may consider supporting children’s psychological needs in the physical activity environment through provision of activity choice, open-ended questions, and positive constructive feedback.

## Background

Well-being is a key indicator of health and refers to an individual’s optimal psychological functioning [1]. Globally, approximately 80% of school youth do not meet the World Health Organization’s (WHO) moderate-to-vigorous physical activity (MVPA) guidelines for health [2], with similar figures (i.e. 82%) reported among Irish children of low socio-economic status (SES) [3]. The adverse health effects of physical inactivity are well established [2], and given studies [4] show that childhood physical activity behaviours track into adulthood, these low figures are of public health concern. Hence, evidence-based physical activity interventions are needed and specifically with children of low socio-economic status (SES) who are at even increased risk of reduced health and well-being compared to the general population [5].

Theory-based physical activity interventions can highlight the psychological and social processes that underlie children’s health and behaviour change [6]. While many school-based physical activity interventions have adopted a theory in their design, few studies have included theoretical constructs related to psychological needs and motivation in their analyses [6, 7]. As such, there remains limited information on the psychosocial mechanisms responsible for improving children’s physical activity levels and well-being [6, 7]. To advance physical activity and well-being promotion it has been proposed that psychosocial variables be included in all aspects of the design and analyses of interventions [6, 8].

To explore behaviour change processes, researchers have applied constructs from self-determination theory (SDT) [1, 9]. Collectively, sub-theories within SDT specify that the satisfaction of humans’ psychological needs for competence (i.e. a sense of effectiveness within environment), autonomy (i.e. volitional behaviour) and relatedness (i.e. belongingness) are essential for optimal motivation, health behaviour and well-being. In support of SDT hypotheses, children’s physical activity has consistently been predicted by autonomous motivation [12], and in some cases, well-being has been predicted by physical activity contexts that satisfy children’s psychological needs [13, 14]. However, SDT has received limited application in school-based intervention studies.

The aim of SDT-informed interventions is to engender needs-supportive social conditions wherein enhancement of physical activity and well-being is realised indirectly through needs-support and satisfaction, and autonomous motivation [10, 11] (see SDT model for health interventions [11]). One validated SDT-informed intervention technique is needs-supportive teaching techniques utilised by intervention deliverers (e.g. school teachers) who can be trained to offer participants physical activity choices (i.e. autonomy support), provide positive instructional feedback (i.e. competence support) and develop a supportive relationship (i.e. relatedness support) [16]. In five school-based intervention studies, participants have been receptive to such techniques by reporting enhanced perceptions of needs-support [15, 17–20]. However, all but one [15] of those studies was with pre-adolescent children (6–12 years), and none were from areas of low SES––a group at risk for reduced well-being [5]. Furthermore, existing studies have either excluded the well-being [17–20] or needs satisfaction [15] components of SDT in their model. Considering the evidence collectively, it is unclear if needs-supportive techniques can exert an influence on each variable on SDT’s causal chain (i.e. autonomy-support, needs satisfaction, motivational regulation) and whether facilitation of those processes predict improved physical activity and well-being among children of low SES.

In response to the outlined limitations, a 10-week intervention called the Healthy Choices Programme (HCP) was developed for 8–9-year-old children of low SES. The programme’s content, delivery and analyses were consistent with SDT hypotheses [9]. The intervention sought to enhance children’s physical activity and well-being through providing needs-supportive teacher training to the delivering sport student volunteers and classroom teachers. The aim was to determine the effect of the HCP through modelling a process that linked autonomy-support, needs satisfaction and intrinsic motivation with physical activity and well-being.

### Study hypotheses

The first hypothesis was that the HCP would increase the intervention group’s perceptions of autonomy-support from their teachers in comparison to a control group (hypothesis 1 (H1)). The second (H2) and third (H3) hypotheses were that intervention group’s needs satisfaction and intrinsic motivation would increase through the mediation of autonomy support. Lastly, hypotheses four (H4) and five (H5) were that the intervention would, respectively, indirectly enhance MVPA and well-being, through the autonomy support, needs satisfaction and intrinsic motivation sequential pathway [11].

## Methods

### Design, Inclusion Criteria, Recruitment Setting and Procedure

The reporting of the HCP adhered to the Transparent Reporting of Evaluations with Non-Randomised Designs (TREND) statement [21] and was registered on Research Registry (trial number 2852). Following approval from Ulster University’s Research Ethics Committee, two schools from Northern Ireland (NI) were identified for a 2 (groups) ×  2 (time-points) wait-list controlled trial. This entailed purposively selecting the intervention and control groups and staggering the delivery of the HCP across two school semesters whilst collecting data at the same time ([22] see Fig. 4). To reduce the potential for contamination, the control school Principal delayed announcement of the HCP until the following school semester, and both schools were unaware of their school’s data being compared during the intervention.

An inclusion criterion was based on the Multiple Deprivation Measure in Northern Ireland [23]. This index has seven domains of socio-economic deprivation including income, services and crime. Having identified schools of low SES on the measure, two schools with likewise demographics (i.e. mixed gender, urban, size) were approached for recruitment. Both school Principals agreed and invited all Primary five pupils to participate. Participant assent and parental consent were gained prior to conducting the research.

A group of trained researchers conducted baseline (week 0) and post-intervention (week 11) measurements (discussed below) with the pupils under quiet classroom conditions. The classroom teacher was present at all times.

### Intervention

The HCP was delivered for 2 h and 15 min each week during school curriculum time for a 10-week period (i.e. 22.5 h of instruction in total). The intervention was in addition to general physical education classes and included (i) weekly hour-long practical sessions delivered by a trained sport student volunteer in tandem with, and under the supervision of classroom teacher and (ii) a ‘Daily Mile’ that involved the classroom teacher leading a 15-min walk every school day. SDT [1, 9] informed several aspects of the programme described below.

The weekly sessions consisted of a series of active discussions and physical tasks that contained messages around the health benefits of physical activity. The student volunteers received a teaching resource detailing language and techniques consistent with needs-supportive tenets in SDT [16], e.g. ‘acknowledge the activities were challenging and congratulate the children for trying their best’. Likewise, the classroom teachers also received a teaching resource including the above language and walking activities that would facilitate autonomy-choice for the children. For example, the ‘mirror image’ activity entailed walking partners completing the Daily Mile in tandem with a choice to mirror each other’s movements.

**Fig. 1:**
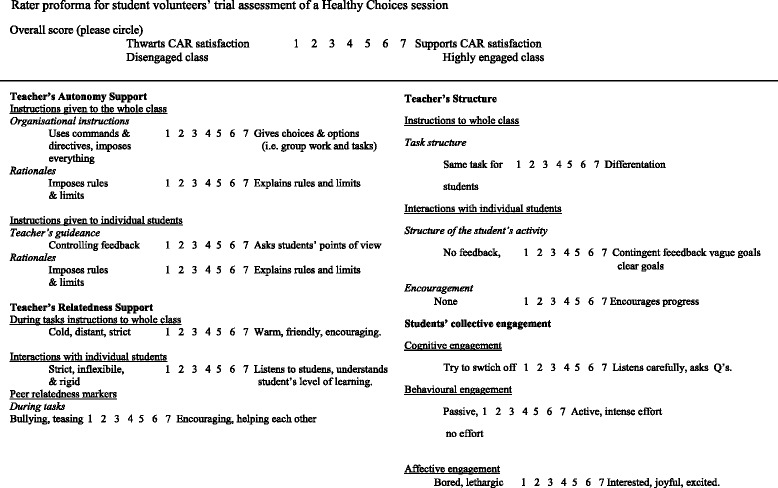
Rater proforma for student volunteers’ trial assessment of a Healthy Choices Programme session

**Fig. 2:**
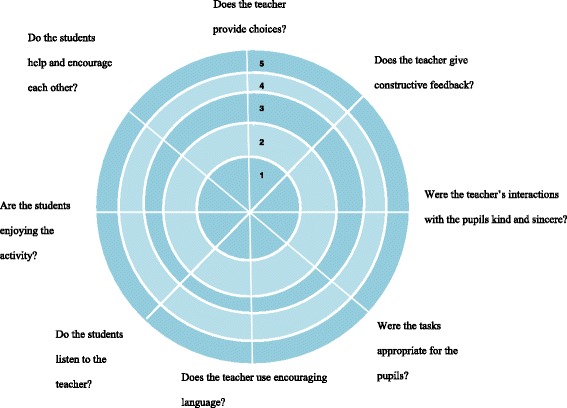
Teaching needs-supportive rater proforma used in student volunteer training

Student volunteers completed a two-day SDT training programme. The training was focused on facilitating the student volunteers’ understanding of a needs-supportive instructional style [16]. Their training included a discussion regarding the students’ experiences of Duda’s [24] empowering vs disempowering climate and a video evaluation of an authoritative-command vs autonomy-supportive teaching style using a rater proforma (see Fig. 1). The students were then presented with vignettes in which children were in need of competence or autonomy-support and were required to produce needs-supportive techniques to enhance engagement. Finally, the students completed a peer-teaching quality assessment of a Healthy Choices Programme session and were assessed in line with an adapted version of Reeve et al.’s [16] teacher observation sheet (see Fig. 2). In the case where improvement was recommended, the student volunteer was asked to reassess their understanding of the aims of the HCP and to engage the vignettes they encountered during training.

The classroom teachers completed a one-day training event in which they were guided on an autonomy-supportive teaching style during the Daily Mile and supervision of the weekly sessions. To link an autonomy-supportive teaching style with relevant teaching practices in the Northern Ireland Key Stage Two Curriculum [25], ‘active learning’ techniques were used. Active learning entails creating a learner-centred environment, in which the children are encouraged to participate in the direction of a lesson through questions, activity choice and feedback [25]. The teachers were asked to develop active learning techniques they could utilise throughout the weekly sessions and Daily Mile (e.g. use of questions, positive feedback, allowing the students to choose content).

In addition, to enhance the children’s relatedness support, parents and/or guardians participated in an insight afternoon. Through consultation, it was decided to update the parents on the HCP through information flyers and videos uploaded to the school’s online ‘parent space’.

### Outcomes

Objective MVPA during school days (i.e. Monday to Friday) and school hours (i.e. 9 am–3 pm, see [26] for time category classification) was measured using Actigraph accelerometers (GT3X and GT1M, Pensacola). The accelerometers were fitted onto the children’s waists with an elasticated belt and positioned on the midaxillary line above the right hip. The devices recorded data in 5 s epochs, a valid capturing period for 8–9-year-old children’s movement [27]. Wells et al.’s [28] wear-time criterion was applied, including at least 8 h wear per-day for a minimum of three weekdays. Children meeting the criteria at both time-points were selected as the ‘valid sample’. Time spent in health-enhancing MVPA intensities [1] were calculated using Evenson cut points [29] deemed the most valid and reliable for 8–9-year-old children [27]. Accelerometer counts of < 20 min of consecutive zeroes, or > 15,000 were removed, as they are considered biologically implausible [27]. For analyses, one variable reflecting the children’s average school-day MVPA was created.

Well-being was measured using the 7-day recall Kidscreen-27 questionnaire [30]. Kidscreen-27 has demonstrated excellent psychometric properties with children aged 8–18 [30] and was recently validated with Irish children of low SES [31]. Kidscreen-27 assesses seven physical, social and psychological well-being dimensions [31], and for analyses, a single variable reflecting the total of the 27-items was created.

To assess the degree to which the children felt their teachers supported their need for autonomy, a modified version of Standage, Duda and Ntoumanis’s [32] Physical Education (PE)-adapted Learning Climate Questionnaire was employed. As the HCP involved physical activity outside of PE (i.e. through the Daily Mile and weekly sessions), the items were modified to reflect autonomy-support during *physical activity classes*. The scale included six items and responses preceded with the stem: ‘In physical activity classes my teacher…’, and were scored using a 7-point Likert scale ranging from ‘strongly disagree’ to ‘strongly agree’. A confirmatory factor analysis (CFA) revealed support for a single latent factor (*χ*2 = 13.961 (9) *p* = .124; CFI = .947; TLI = .912; RMSEA = .063). A scale total was created for analyses.

Children’s perceptions of psychological needs satisfaction (i.e. autonomy, competence and social relatedness) in the context of physical activity were assessed using an age-appropriate questionnaire [33]. The questionnaire included 18 items scored a 5-point Likert scale ranging from ‘not like me at all’ to ‘really like me’ and encompassed three 6-item subscales for autonomy, competence and relatedness. After the omission of the two negatively worded items (item 4 autonomy, and item 12 competence), a CFA within the sample revealed a good-fitting three-factor model with covariance paths between the latent variables (*χ*2 = 152.789 (99) *p* = .000; CFI = .920; TLI = .903; RMSEA = .065). A total needs satisfaction variable was created for analyses.

Four dimensions of SDT’s motivation continuum were measured using an age-appropriate questionnaire [33]. The questionnaire included 12 items encompassing four 3-item motivation subscales (i.e. intrinsic motivation, identified regulation, introjected regulation and external regulation) answered on a 5-point likert scale ranging from ‘not like me at all’ to ‘really like me’. A four-factor model consisting of two latent co-varying factors (i.e. identified with intrinsic motivation and introjected regulation with external regulation) yielded an unacceptable fit. However, correlating three items (i.e. item in 1 intrinsic motivation with item 2, and 10 in identified regulation; and item 11 in introjected regulation with item 12 external regulation) theoretically aligned with Ryan and Deci’s [9] conception of autonomous and controlled motivation in SDT, subsequently yielded an acceptable fit (*χ*2 = 81.982 (45), *p* = .001; CFI = .937; TLI = .907; RMSEA = .077). Scale totals for each dimension were created.

### Data Management

Raw data from each individual questionnaire was manually inputted into SPSS (Version 22; IBM Corp., NY). Ten percent of questionnaires were checked as a quality assurance procedure. The expectation maximisation algorithm was conducted on each independent scale to estimate missing data after Little’s Missing Completely at Random test confirmed that the data was missing at random on both time-points (*p* > .05).

### Statistical Analyses

**Fig. 3:**
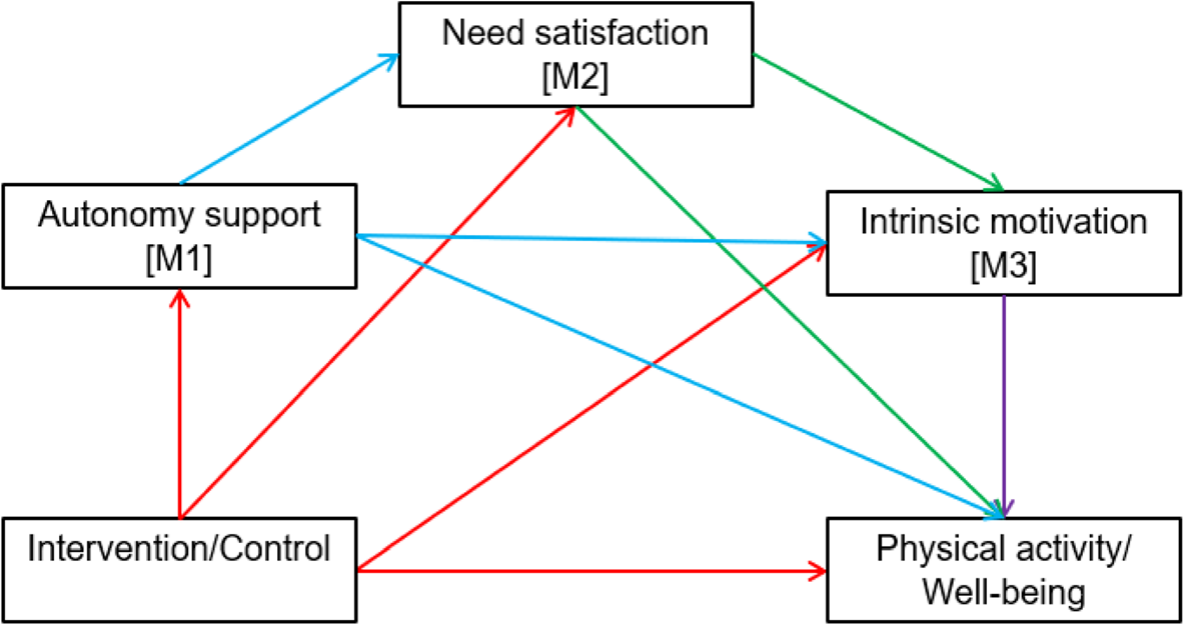
Hypothesised model 1 (physical activity) and 2 (well-being) with three mediators specifying the one direct and seven indirect effects of *X* (intervention) on *Y* (well-being)

Two models subscribing to Fortier, Duda, Guerin and Teixeira’s [11] SDT model for health interventions were specified. The aim of testing the models was to determine if changes in the children’s perceptions of autonomy-support (from teachers) would indirectly affect changes on the primary outcomes of MVPA (model 1) and well-being (model 2) through needs satisfaction and intrinsic motivation (see Fig. 3).

The independent variable (*X*) was coded as a dichotomous variable (control = 0 and intervention = 1). Difference scores were created by subtracting post-intervention scores from baseline. MVPA and well-being were coded as dependent variables (*Y*). Mediator 1 (M1) refers to autonomy-support, mediator 2 (M2) as needs satisfaction, and mediator 3 (M3) as intrinsic motivation. Intrinsic motivation was selected as M3 because it is assumed and has been empirically found to yield the most adaptive outcomes in terms of increasing MVPA in children [3] and well-being [13] (see Additional file 1 wherein identified regulation, introjected regulation and external regulation were selected as M3).

The procedures described by Hayes [34] were used, testing one direct effect between *X* on *Y* ($\overset{\acute{\mkern6mu}}{c}$) and seven singular or serial indirect effects between *X* on *Y* through M1, M2 and M3. Hayes’ model also examines three direct and three indirect effects between *X* on the three mediators. The results can confirm if the effect of *X* (intervention) on *Y* (outcomes) is either (i) not significant, (ii) fully explained by the mediators (i.e. full mediation), (iii) partially explained through the mediators (i.e. partial mediation) or (iv) indirectly explained through the mediators (i.e. indirect effects) [35].

Two figures were produced specifying beta (β) coefficient values for each direct path and *r*^*2*^ values related to the proportion of total variance predicted in model 1 and model 2. A table was created to detail the completely standardised effect sizes and confidence intervals for each of the seven indirect effects of the intervention on the dependent variables. If confidence intervals did not cross zero, the indirect relationship was interpreted as statistically significant [36]. For improved accuracy, the models were tested with 5000 bootstrap samples [35]. Analyses were conducted using Hayes’ [37] PROCESS macro for SPSS (Version 22; IBM Corp, NY).

## Results

**Fig. 4:**
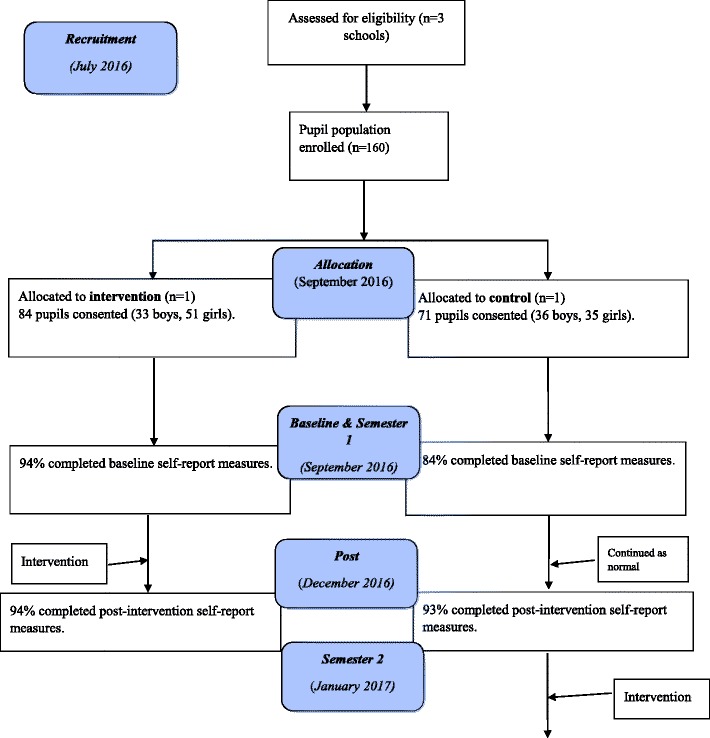
Flow diagram describing the design and flow of participants through the Healthy Choices Programme

The recruitment dates, sample characteristics, flow of participants and attrition rates through each stage of the study are presented in Fig. 4. The total sample size was 155 children, comprising 72 boys and 82 girls with a mean age of 8.7 years (SD = .50). The intervention group included 84 (54.2%) children, and the control group included 71 (45.8%). Table 1 details the mean and standard deviation scores for each outcome variable at baseline and post-intervention. On average, a 10% attrition rate was found at baseline and 7% at post-intervention.

**Table 1:** Descriptive statistics for each outcome measure at baseline and post-intervention

Variables	Intervention *M* (*n*), SD	Control *M* (*n*), SD
Accelerometer - MVPA		
Baseline	21.06(67) 6.24	23.48 (51) 7.48
Post	24.91 (46) 7.48*	19.50 (26) 8.20
K-27 total		
Baseline	115.12 (76) 16.59	110.78 (56) 15.37
Post	118.88 (76) 15.11	112.40 (56) 14.51
Autonomy support		
Baseline	31.02 (76) 6.03	28.90 (56) 6.48
Post	33.68 (76) 7.24*	28.51 (56) 6.20
Autonomy satisfaction		
Baseline	16.99 (76) 4.87	17.62 (56) 4.51
Post	19.14 (76) 4.03*	17.85 (56) 4.20
Competence satisfaction		
Baseline	18.27 (76) 5.25	18.47 (56) 4.53
Post	19.30 (76) 4.15	18.66 (56) 4.40
Relatedness satisfaction		
Baseline	23.38 (76) 5.53	22.12 (56) 6.38
Post	24.59 (76) 5.68	22.31 (56) 6.55
Total needs satisfaction		
Baseline	58.04 (76) 13.44	58.22 (56) 12.82
Post	63.05 (76) 11.68	58.82 (56) 11.58
Intrinsic motivation		
Baseline	12.53 (76) 3.34	12.23 (56) 3.98
Post	13.47 (76) 2.85	13.01 (56) 2.97
Identified regulation		
Baseline	11.31 (76) 3.41	10.81 (56) 3.17
Post	12.54 (76) 2.91	11.55 (56) 2.98
Introjected regulation		
Baseline	8.78 (76) 3.34	8.88 (56) 3.58
Post	9.35 (76) 3.71	9.10 (56) .3.74
External regulation		
Baseline	7.52 (76) 3.46	7.37 (56) 3.12
Post	6.80 (76) 3.56	6.32 (56) 2.91

*M* mean, *n* sample size, *SD* standard deviation, *K-27* Kidscreen-27

*Significant interaction effect for group and time from baseline to post-intervention

### Model 1: MVPA

The results of model 1 confirmed that taking part in the HCP significantly and directly enhanced MVPA (*β* = .45, *p* = .005) and autonomy-support (M1; *β* = .17, *p* = .003). The intervention group’s mean minutes of MVPA increased from 21.06 (SD 6.24) at baseline to 24.91 (SD 7.48) at post-intervention, while the control group’s post-intervention mean minutes (*M* 23.48, SD 7.14) decreased in comparison to their baseline (*M* 19.50, SD 8.20; see Table 1).

When exploring the direct and indirect effects of the intervention on M1, M2 and M3, the results revealed that the direct effect of the intervention on M1 did not in turn influence M2 and M3. However, this was not the case for model 2 (see below), suggesting the null effects were attributable to the reduced sample size in model 1 (*n* = 62) because of non-compliance with accelerometer wear-time criteria.

**Fig. 5:**
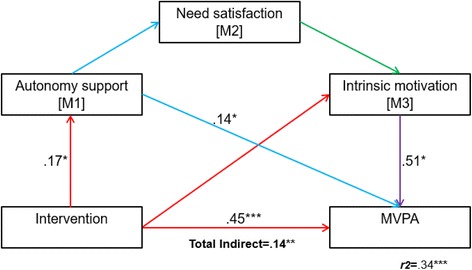
Model 1 (MVPA) findings describing the two singular and one serial indirect effects of the intervention on MVPA

The intervention indirectly enhanced MVPA through singular mediation of autonomy-support (M1; *β* = .14, 95% CI [.010 to .158], *p* < .05), and intrinsic motivation (M3; *β* = .51, 95% CI’s [.000 to .134], *p* = .04). The intervention also indirectly enhanced MVPA through serial mediation of M1 (autonomy support), M2 (needs satisfaction) and M3 (intrinsic motivation) (95% CI [.000 to .023], *p* < .05). In comparison to the variance predicted for the intervention’s direct effect on MVPA alone (*r*^*2*^ = .20), factoring in M1, M2 and M3 resulted in a greater predicted MVPA variance (*p* = .001, *r*^*2*^ = .34). Once controlling for SDT mediators, the direct effect of intervention on MVPA remained, concluding partial mediation (see Fig. 5 for a visual description of model 1 and Table 2 for values for each path).

**Table 2:** Mediation table for intervention effects

Model: dependent variable	Treatment on dependent variable *β* coefficient (*p* value)	Hypothesised mediators	Treatment on mediator *β* coefficient (*p* value)	M1 on M2 and M3 *β* coefficient (*p* value) [95% CI]	M2 on M3 *β* coefficient (*p* value) [95% CI]	Treatment > M1 > dependent variable Effect [95% CI]	Treatment > M2 > dependent variable Effect [95% CI]	Treatment > M3 > dependent variable Effect [95% CI]	Treatment > M1 > M2 > dependent variable Effect [95% CI]	Treatment > M1 > M3 > dependent variable Effect [95% CI]	Treatment > M2 > M3 > dependent variable Effect [95% CI]	Treatment > M1 > M2 > M3 > dependent variable Effect [95% CI]	Total sum of all the indirect effects Effect [95% CI
1. Physical activity	.45 (.00***)	Autonomy-support (M1)	.17 (.04*)	n/a	n/a	.06 [.012 to .163]*	.01 [− .015 to .087]	*.04 [.000 to .134]*	.01 [− .002 to .059]	− .00 [− .036 to .011]	.00 [− .004 to .037]	.004 [.000 to .023]*	.14 [.047 to .267]*
		Needs satisfaction (M2)	.15 (.08)	.42 (.05) [− .004 to .846]	n/a								
		Intrinsic motivation (M3)	.02 (.80)	− .01 (.70) [− .118 to .084]	.04, (.12) [− .012 to .105]								
2. Well-being	.07 (.42)	Autonomy-support (M1)	.17. (04*)	n/a	n/a	.03 [.004 to .104]*	.03 [− .015 to .099]	− .00 [− .038 to .011]	.01 [.003 to .054]*	.00 [− .002 to .009]	.00 [− .005 to .019]	.00 [− .002 to .007]	*.09 [.010 to .194* ]*
		Needs satisfaction (M2)	.15 (.08)	.43 (.00***) [.186 to .685]	n/a								
		Intrinsic motivation (M3)	.02 (80)	.03 (.29) [− .035 to .113]	.11 (.00***) [.066 to .165]								

*Note:* Control and intervention groups were coded as 0 and 1, respectively

*CI* lower and upper confidence intervals, *n/a* non-applicable, italic type confidence intervals indicate a significance at *p* < .05 because the CIs do not include zero

*p < .05, **p < .01, ***p < .001

### Model 2: Well-Being

The results of model 2 confirmed that on its own, the intervention did not directly enhance well-being (*r*^*2*^ = .05, *p* = .42). However, when factoring in the mediators, the intervention indirectly and significantly enhanced well-being (*r*^*2*^ = .21, *p* = .001), through a combination of singular and serial indirect mechanisms outlined below.

When exploring the direct and indirect effects of the intervention on M1, M2 and M3, the results were consistent with Deci and Ryan’s (2000) assumptions. The direct effect of the intervention on autonomy-support (M1, *β* = .17, *p* < .04) resulted in an indirect effect of the intervention on needs satisfaction (M2, *β* = .43, 95% CI [.186 to .685], *p* = .001). Further, and in relation to the sample size reference above for model 1, the increased sample size in model 2 (*n* = 132) resulted in an indirect effect of the intervention on M3 (*β* = .11, 95% CI [.066 to .165], *p* = .001) through M2.

**Fig. 6:**
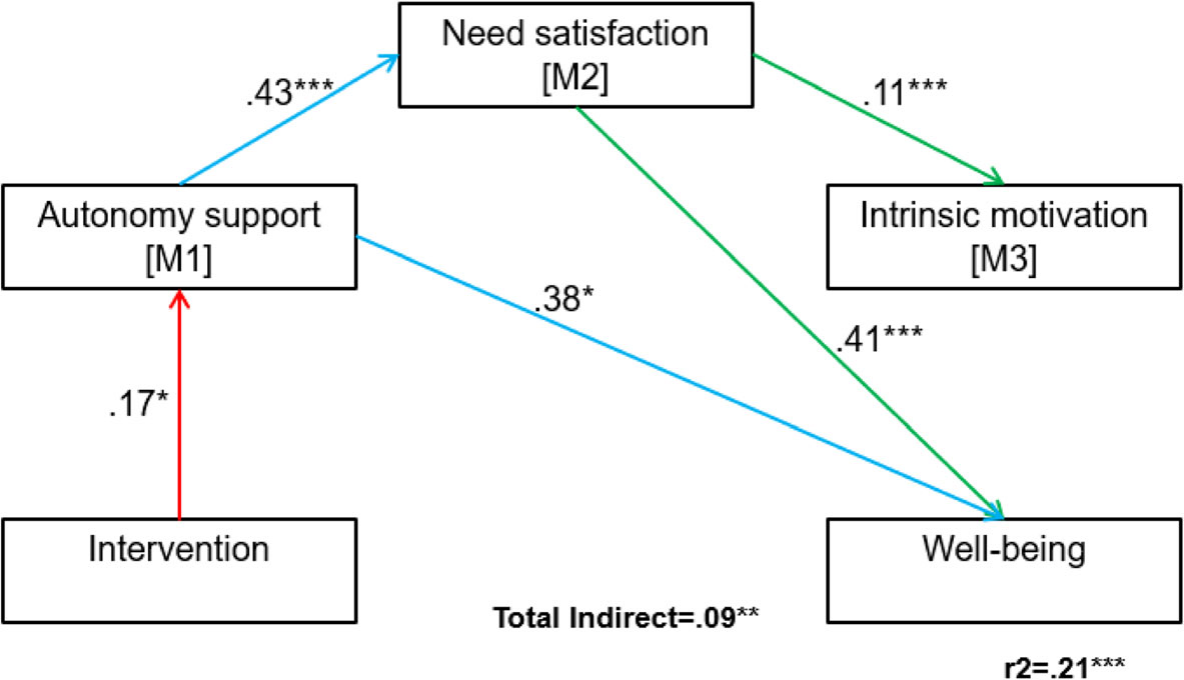
Model 2 (well-being) findings describing the one singular and one serial indirect effects of the intervention on well-being

The intervention indirectly enhanced well-being through autonomy-support (M1, *β* = .38, 95% CI [.004 to .104], *p* = .01) and through autonomy-support and needs satisfaction in serial (*β* = .15, 95% CI [.003 to .054], *p* < .05). There was no significant indirect effect of the intervention through the autonomy support, needs satisfaction and intrinsic motivation sequence (see Table 2 for values for each path and Fig. 6 for a visual model depiction).

## Discussion

This was the first study to apply SDT in the design and analyses of a school-based intervention aimed at enhancing MVPA and well-being among children of low SES. The HCP was designed to enhance children’s perceptions of autonomy-support, needs satisfaction and intrinsic motivation through training sport student volunteers and classroom teachers to utilise needs-supportive teaching principles. The research aim was to test the effect of the HCP on the children’s MVPA and well-being through Fortier et al.’s [11] SDT model for health interventions. The results highlighted a number of psychosocial processes that underlie the mechanisms of MVPA and well-being promotion [6]. In support of H1, the intervention group perceived more support for their need for autonomy than the control group from baseline to post-intervention. Exploring the residual causal sequence revealed that the intervention indirectly enhanced MVPA through partial mediation of autonomy-support, needs satisfaction and intrinsic motivation and indirectly enhanced well-being through autonomy-support and needs satisfaction. These findings indicate that needs-supportive physical activity environments can facilitate positive motivational states, MVPA behaviour and well-being [1, 11]. Ways to advance SDT in health promotion are now discussed.

By training teachers to offer physical activity choice, participatory learning, positive constructive feedback and meaningful rationale during the Daily Mile and supervision weekly sessions, the children’s need for autonomy was enhanced. This finding corroborates Ryan and Deci’s [9] description of needs-supportive social environments and aligns with studies in the PE context [18, 19, 38] wherein pupils have been receptive to their teacher’s modified instructional style. In accordance with SDT hypotheses, in model 2, the direct effect of the intervention on autonomy-support exerted indirect effects on needs satisfaction and intrinsic motivation, confirming hypotheses 2 (H2) and 3 (H3). Support for H2 and H3 provide confirmatory evidence of the mediating role of autonomy-support described in SDT [1], in which the children’s school environment facilitated psychological needs satisfaction and intrinsic motivation for physical activity [39]. As such, our findings are consistent with the trans-contextual model of motivation [39], suggesting that autonomy-support from teachers can transfer its effects to general physical activity motivations.

In comparison to the control group, the intervention group increased their total and MVPA during school days from baseline to post-intervention (i.e. 4.49 min improvement). Whilst regular MVPA is essential for children’s health [2], many school teachers indicate time as a barrier for behaviour change [40]. This study highlighted that integrating basic, time-efficient, and physical activities into the school day can have a meaningful impact on children’s behaviour change, suggesting that educators consider completing curriculum-based activities with physical activity [40]. Moreover, the psychosocial processes reported for these effects can inform future health promotion efforts. Consistent with SDT, the MVPA variance was explained through partial mediation of autonomy-support, needs satisfaction and intrinsic motivation (H4). Support for H4 provides evidence congruent with a meta-analysis of 46 studies [12], suggesting that pre-adolescent children’s physical activity can be enhanced and is most strongly regulated through autonomous intrinsic motivational states rather than extrinsic motives. However, there was a degree of variance unexplained by SDT constructs in model 1. The lack of full mediation through SDT mediators is unsurprising given that the Integrated Behaviour Change Model [41], among other dual-process models [42], denotes unconscious psychological processes beyond intentional motivations that provide schema for children’s physical activity (e.g. affective responses, see [42] for a review). Future research may consider testing SDT alongside assumptions within or alongside validated dual-process models [41, 42] to improve the prediction and enhancement of physical activity behaviours in children.

The HCP did not exert a significant direct effect on well-being, supporting conclusions in a recent systematic review of school-based physical activity interventions [43] and randomised controlled trial designed to increase well-being [44]. However, the HCP had an indirect effect on well-being through autonomy-support and needs satisfaction, confirming H5. This finding, coupled with the results of a school-based screen-time intervention incorporating SDT [45], supports the conceptualisation of well-being from a eudemonic perspective [1], in which well-being is realised through the social environment providing support for one’s psychological needs [46].

Furthermore, the indirect effect of the intervention on well-being through autonomy-support and needs satisfaction reinforces previous research that documented a positive correlation between needs satisfaction during physical activity and well-being [13, 14], indicating that needs satisfaction at a domain level (i.e. physical activity) may transfer its effects to well-being at a global day-to-day level. In addition, while previous research [47] has reported a direct unidimensional relationship between physical activity and well-being, the indirect effects found in the present study suggest a more nuanced association [7]. The psychosocial explanation that physical activity contexts provide an opportunity for social belongingness (i.e. relatedness), environmental mastery (i.e. competence) and volition (i.e. autonomy) was evidenced to facilitate well-being among children of low SES. When aiming to enhance well-being through physical activity, researchers and practitioners may consider modifying the social climate through offering psychological needs-support rather than just the behaviour alone [14].

### Generalisability and Limitations

The design of this study was specific to children in the school setting. Therefore, adaptation and use of needs-supportive techniques for other populations (e.g. adults) and contexts (e.g. online) may refer to a recent review on needs-supportive physical activity communication [48]. While the authors followed available methodological guidance [40] by maintaining communication with the delivering teachers and student volunteers, including revisiting the aims of the SDT principles applied [48], there was a lack of empirical fidelity data upon which to conclude on the efficacy of the study fidelity. We refer the reader to a recent theoretical fidelity evaluation study of a likewise SDT programme [49] for addressing such issues in future work. Moreover, it was not possible to conduct a follow-up to test whether the effects reported maintained longitudinally a recognised limitation of the waiting list design [22]. While all efforts were ensured to reduce the potential for contamination, the design of this study would have been improved through a clustered randomised control trial design comprising additional participants.

## Conclusions

A key strength of the current study was the design, application and analyses of SDT-informed needs-supportive teaching techniques. This study demonstrated that the HCP enhanced MVPA partially through increasing the children’s perceptions of autonomy-support, needs satisfaction and intrinsic motivation. Well-being was also indirectly enhanced through improvements in autonomy-support and needs satisfaction. The indirect effects of the HCP highlighted motivational and needs-supportive mechanisms underpinning children’s MVPA participation and well-being. As such, the practical implications of this study can guide researchers and practitioners towards modifying the social environment in which physical activity is experienced through utilising needs-supportive teaching principles [48]. To build on the findings from this research, further work may consider conducting a clustered RCT incorporating the recommended methodologies to explore if such changes can be maintained longitudinally. Such work could examine the influence of needs-support provided to the children by their teachers, student volunteers and parents, who all contributed to the intervention. Overall, the HCP is a theory-driven study that can advance health promotion in the school setting.

## Additional file

Additional file 1: Serial mediation models treating identified regulation, introjected regulation and extrinsic motivation as mediator 3 (M3). (DOCX 30 kb)

## Abbreviations

HCP: Healthy Choices Programme PE Physical education
WHO: World Health Organization MVPA Moderate-to-vigorous physical activity SES Socio-economic status SDT Self-determination theory
