# Supplementary material for: The Effect of a School-Based Intervention on Physical Activity and Well-Being: a Non-Randomised Controlled Trial with Children of Low Socio-Economic Status
Source: Sports Med Open. 2018 Apr 20;4:16. doi: 10.1186/s40798-018-0129-0 (PMC5910444; doi:10.1186/s40798-018-0129-0)
Supplement: Supplementary file 1 — Serial mediation models treating identified regulation, introjected regulation and extrinsic motivation as mediator 3 (M3). (DOCX 30 kb) [file 40798_2018_129_MOESM1_ESM.docx]

Additional File 2: Serial mediation models treating identified regulation, introjected regulation and extrinsic motivation as mediator 3 (M3).

| **Model: *dependent variable;*** | **Treatment on dependent variable** β coefficient (p value) | **Hypothesised Mediators** | **Treatment on mediator** β coefficient (p value) | **M1 on M2 & M3** β coefficient (p value) [95% CI] | **M2 on M3** β coefficient (p value) [95% CI] | **Treatment > M1 > dependent variable**  Effect [95% CI] | **Treatment > M2 > dependent variable**  Effect [95% CI] | **Treatment > M3 > dependent variable**  Effect [95% CI] | **Treatment > M1 > M2 > dependent variable** Effect [95% CI] | **Treatment > M1 > M3 > dependent variable** Effect [95% CI] | **Treatment > M2 > M3 > dependent variable** Effect [95% CI] | **Treatment > M1 > M2 > M3 > dependent variable** Effect [95% CI] |
| --- | --- | --- | --- | --- | --- | --- | --- | --- | --- | --- | --- | --- |
| ***Physical***  ***Activity;*** | .45 (.00***) | Autonomy-support (M1) | .17 (.04*) | n/a | n/a | **.06 [.012 to .163]*** | .01 [-.015 to .087] | .000 [-.043 to .037] | .01 [-.002 to .059] | .00 [-.017 to .017] | .00 [-.016 to .014]] | .00 [-.010 to .009] |
|  |  | Needs satisfaction (M2) | .15 (.08) | .42 (.05)  [-.004 to .846] | n/a |  |  |  |  |  |  |  |
|  |  | Identified regulation (M3) | .04 (.95) | .00 (.87)  [-.087 to .102] | **.06, (.03*)  [.004 to .117]** |  |  |  |  |  |  |  |
| 2:  ***Well-being*** | .07 (.42) | Autonomy-support (M1) | .17, (04*) | n/a | n/a | **.03 [.004 to .104]*** | .03 [-.015 to .099] | .00 [-.015 to .040] | **.01 [.003 to .054]*** | .00 [-.023 to .108] | .00 [-.029 to .002] | .00 [-.013 to .000] |
|  |  | Needs satisfaction (M2) | .15.(08) | .**43 (.00***)** **[.186 to .685]** | n/a |  |  |  |  |  |  |  |
|  |  | Identified regulation (M3) | .04 (.80) | .03 (.29) [-.035 to .113] | **.11 (.00***) [.066 to .165]** |  |  |  |  |  |  |  |

| **Model: *dependent variable;*** | **Treatment on dependent variable** β coefficient (p value) | **Hypothesised Mediators** | **Treatment on mediator** β coefficient (p value) | **M1 on M2 & M3** β coefficient (p value) [95% CI] | **M2 on M3** β coefficient (p value) [95% CI] | **Treatment > M1 > dependent variable**  Effect [95% CI] | **Treatment > M2 > dependent variable**  Effect [95% CI] | **Treatment > M3 > dependent variable**  Effect [95% CI] | **Treatment > M1 > M2 > dependent variable** Effect [95% CI] | **Treatment > M1 > M3 > dependent variable** Effect [95% CI] | **Treatment > M2 > M3 > dependent variable** Effect [95% CI] | **Treatment > M1 > M2 > M3 > dependent variable** Effect [95% CI] |
| --- | --- | --- | --- | --- | --- | --- | --- | --- | --- | --- | --- | --- |
| ***Physical***  ***Activity;*** | .45 (.00***) | Autonomy-support (M1) | .17 (.04*) | n/a | n/a | **.06 [.012 to .163]*** | .01 [-.015 to .087] | .01 [-.107 to .012] | .01 [-.002 to .059] | .00 [-.003 to .047] | .00 [-.004 to .005] | .00 [-.003 to .004] |
|  |  | Needs satisfaction (M2) | .15 (.08) | .42 (.05)  [-.004 to .846] | n/a |  |  |  |  |  |  |  |
|  |  | Introjected regulation (M3) | -.15 (.46) | -.04 (.51) [-.161 to .083] | .00 (.99) [-.073 to .073] |  |  |  |  |  |  |  |
| 2:  ***Well-being*** | .07 (.42) | Autonomy-support (M1) | .17, (04*) | n/a | n/a | **.03 [.004 to .104]*** | .03 [-.015 to .099] | .00 [-.021 to .031] | **.01 [.003 to .054]*** | .00 [-.007 to .003] | .00 [-.000 to .018] | .00 [-.000 to .018] |
|  |  | Needs satisfaction (M2) | .15.(08) | .**43 (.00***)** **[.186 to .685]** | n/a |  |  |  |  |  |  |  |
|  |  | Introjected regulation (M3) | -.15 (.46) | -.04 (.51) [-.161 to .083] | .00 (.99) [-.073 to .073] |  |  |  |  |  |  |  |

| **Model: *dependent variable;*** | **Treatment on dependent variable** β coefficient (p value) | **Hypothesised Mediators** | **Treatment on mediator** β coefficient (p value) | **M1 on M2 & M3** β coefficient (p value) [95% CI] | **M2 on M3** β coefficient (p value) [95% CI] | **Treatment > M1 > dependent variable**  Effect [95% CI] | **Treatment > M2 > dependent variable**  Effect [95% CI] | **Treatment > M3 > dependent variable**  Effect [95% CI] | **Treatment > M1 > M2 > dependent variable** Effect [95% CI] | **Treatment > M1 > M3 > dependent variable** Effect [95% CI] | **Treatment > M2 > M3 > dependent variable** Effect [95% CI] | **Treatment > M1 > M2 > M3 > dependent variable** Effect [95% CI] |
| --- | --- | --- | --- | --- | --- | --- | --- | --- | --- | --- | --- | --- |
| ***Physical***  ***Activity;*** | .45 (.00***) | Autonomy-support (M1) | .17 (.04*) | n/a | n/a | **.06 [.012 to .163]*** | .01 [-.015 to .087] | -.00 [-.017 to .041] | .01 [-.002 to .059] | -.00 [-.012 to .006] | .00 [-.002 to .016] | .00 [-.017 to .041] |
|  |  | Needs satisfaction (M2) | .15 (.08) | .42 (.05)  [-.004 to .846] | n/a |  |  |  |  |  |  |  |
|  |  | External regulation (M3) | .35 (.80) | -.00 (.93) [-.172 to .166] | .06 (.19) [-.034 to .162] |  |  |  |  |  |  |  |
| 2:  ***Well-being*** | .07 (.42) | Autonomy-support (M1) | .17, (04*) | n/a | n/a | **.03 [.004 to .104]*** | .03 [-.015 to .099] | .00 [-.011 to .025] | **.01 [.003 to .054]*** | .00 [-.009 to .000] | .00 [-.000 to .011] | .00 [-.000 to .005] |
|  |  | Needs satisfaction (M2) | .15.(08) | .**43 (.00***)** **[.186 to .685]** | n/a |  |  |  |  |  |  |  |
|  |  | External regulation (M3) | .35 (.80) | -.00 (.93) [-.172 to .166] | .06 (.19) [-.034 to .162] |  |  |  |  |  |  |  |

**Note:** Control and intervention groups were coded as 0 and 1, respectively. *=p<.05; **=p<.01, ***=p<.001; CI = lower and upper confidence intervals; n/a = non applicable; **bold type** confidence intervals indicate a significance at p<.05 because the CIs do not include zero.

Note: Control and intervention groups were coded as 0 and 1, respectively. *=p<.05; **=p<.01, ***=p<.001; CI = lower and upper confidence intervals; n/a = non applicable; **bold type** confidence intervals indicate a significance at p<.05 because the CIs do not include zero.
